# Locomotor-Respiratory Coupling in Wheelchair Racing Athletes: A Pilot Study

**DOI:** 10.3389/fphys.2016.00011

**Published:** 2016-01-29

**Authors:** Claudio Perret, Martin Wenger, Christof A. Leicht, Victoria L. Goosey-Tolfrey

**Affiliations:** ^1^Institute of Sports Medicine, Swiss Paraplegic CentreNottwil, Switzerland; ^2^European Research Group in Disability Sport; ^3^Institute of Sport Science, Faculty of Human Sciences, University of BernBern, Switzerland; ^4^The Peter Harrison Centre for Disability Sport, School of Sport, Exercise and Health Sciences, Loughborough UniversityLoughborough, UK

**Keywords:** spinal cord injury, elite sports, exercise, respiratory muscles, push frequency

## Abstract

**Purpose:** In wheelchair racing, respiratory muscles of the rib cage are concomitantly involved in non-ventilatory functions during wheelchair propulsion. However, the relationship between locomotor-respiratory coupling (LRC: the ratio between push and breathing frequency), respiratory parameters and work efficiency is unknown. Therefore, the aim of the present study was to investigate the LRC in wheelchair racers over different race distances.

**Methods:** Eight trained and experienced wheelchair racers completed three time-trials over the distances of 400, 800, and 5000 m on a training roller in randomized order. During the time trials, ventilatory and gas exchange variables as well as push frequency were continuously registered to determine possible LRC strategies.

**Results:** Four different coupling ratios were identified, namely 1:1; 2:1, 3:1 as well as a 1:1/2:1 alternating type, respectively. The 2:1 coupling was the most dominant type. The 1:1/2:1 alternating coupling type was found predominantly during the 400 m time-trial. Longer race distances tended to result in an increased coupling ratio (e.g., from 1:1 toward 2:1), and an increase in coupling ratio toward a more efficient respiration was found over the 5000 m distance. A significant correlation (*r* = 0.80, *p* < 0.05) between respiratory frequency and the respiratory equivalent for oxygen was found for the 400 m and the 800 m time-trials.

**Conclusions:** These findings suggest that a higher coupling ratio indicates enhanced breathing work efficiency with a concomitant deeper and slower respiration during wheelchair racing. Thus, the selection of an appropriate LRC strategy may help to optimize wheelchair racing performance.

## Introduction

Wheelchair athletes are continually searching to optimize their push frequency strategies (Goosey et al., 2000). In wheelchair racing the movement pattern of the upper extremities follows a repetitive cyclic nature yet the arm movement pattern is not constrained and the athletes are “free” in their technique (Van der Woude et al., 1989). It is evident from wheelchair race disciplines that athletes choose a wide range of push frequencies (Goosey et al., 1997, 1998; Goosey and Campbell, 1998). For example, some athletes utilize a slow constant push strategy (Goosey et al., 2000) whilst others select a sequenced type of pushing such as three to four forceful pushes followed by a rest period (Hutzler, 1998). It is not clear what determines these differences in push strategy and how they are affected by race discipline duration. Possibly, the coordination between locomotion and respiration (locomotor-respiratory coupling; LRC) during exercise could help explain these observations.

The existence of LRC has already been described for animals and humans some decades ago by Bramble and Carrier (1983). The advantage of the application of an adequate LRC strategy seems to be a more efficient respiration, leading to a reduction in oxygen consumption as shown for cycling by Bernasconi and Kohl (1993). Thus, an efficient LRC strategy might play an important role in optimizing exercise performance by means of a coupling strategy which reduces the oxygen consumption to a minimum for a given exercise intensity (O'Halloran et al., 2012). For upper body exercise such as rowing a constant coupling ratio seems to be more efficient compared to a ratio change (Mahler et al., 1991a,b). However, only few studies have investigated the LRC ratio during wheelchair propulsion in a wheelchair sporting context; data that can be gleaned from those studies exploring LRC of wheelchair propulsion are limited due to the methodological limitations of slow propulsive speeds and/or use of able-bodied participants (MacDonald et al., 1992; Amazeen et al., 2001). That said, experienced wheelchair users tended to prefer a 2:1 coupling ratio in their daily wheelchair (Amazeen et al., 2001), which remained constant with increasing submaximal speeds from 1.0 to 1.8 m·s^−1^. Furthermore, whole-number coupling ratios (mostly 4:1, 3:1, and 2:1) were found, which underlines the existence of a LRC strategy during this mode of exercise. The same conclusion can be drawn from findings of Goosey et al. (2000), who were able to show a general coordination of in—and expiration with the time point of hand-wheel contact in wheelchair athletics. It seems that an increasing push frequency results in a significant increase of the coupling ratio toward 3:1 when using a daily wheelchair (Amazeen et al., 2001). On the other hand, both MacDonald et al. (1992) and Fabre et al. (2005) found that the same LRC occurred during hand rim propulsion regardless of push frequency. Nevertheless, it is not clear whether or not this LRC phenomenon occurs during wheelchair racing with speeds up to 8.5 m·s^−1^ and push rates >120 pushes·min^−1^. If so, coaches may be able to train athletes toward an efficient coupling of respiration and locomotion and in turn optimize wheelchair racing performance. Therefore, the aim of the present study was to investigate the LRC in wheelchair racers over different race distances. We hypothesized to find different LRC types over the different race distances and expected a lower coupling ratio due to a more efficient breathing pattern with increasing race distances and lower speeds.

## Materials and methods

### Participants

Eight experienced well trained competitive wheelchair racers from the T53 and T54 racing classification participated in the study. The group comprised of six males and two females, all familiar with the applied testing procedures as such exercise testing interventions were part of their training routine and were performed on a regular basis. All tests were performed in the athletes' own racing wheelchairs with constant tire pressure and self-selected handrim size for each individual and with no abdominal binders worn during the trials.

### Study design

The study was approved by the Local Ethical Committee of the Canton Lucerne and participants gave their written informed consent in accordance with the Declaration of Helsinki before the start of the study. Participants performed a total of four testing sessions at least 48 h apart at the same time of day.

The first session consisted of a ramp exercise test on a treadmill (HP Cosmos, Nussdorf-Traunstein, Germany) to determine peak oxygen consumption (VO_2peak_). Respiratory parameters were measured breath by breath by means of an ergospirometric device (Oxycon Pro, Jäger, Würzburg, Germany). The test started at a speed of 3.9 m·s^−1^ with a treadmill inclination of 2%. Every minute, treadmill speed was increased by 0.28 m·s^−1^ until subjective exhaustion. VO_2peak_ was determined as the highest 15 s average value during the ramp exercise test.

The following tests were performed on a training roller (Reha-Blitz, Uetendorf, Switzerland) in randomized order. After a 10 min warm-up, participants performed a 400, 800, or 5000 m time-trial at race pace. During these trials ventilatory (breathing frequency, tidal volume) and gas exchange (oxygen uptake and its respiratory equivalent) variables as well as push frequency were continuously recorded with the above mentioned ergospirometric device and a video camera (NV-GS 37, Panasonic, Osaka, Japan), respectively. Subsequently, the different coupling types were determined by calculating the ratio between push frequency and respiratory frequency based on 5 s average values, excluding the first 15 s of the tests. Blood lactate was measured enzymatically (Super GL Ambulance, Ruhrtal Labor Technik, Möhnesee, Germany) from a capillary sample from the earlobe immediately following the tests, and physical (overall) and respiratory fatigue were assessed with a visual analog scale.

### Statistical analysis

For data analysis 5 s average values were used and data are presented as mean ± standard deviation. To compare average respiratory and push frequencies between the different race distances a repeated measures one-way ANOVA was performed. Correlation coefficients comparing respiratory frequency with the respiratory equivalent for oxygen as well as with the tidal volume were calculated for each race distance separately according to Spearman using a statistical software package (SPSS, Version 15.0.1). The level of significance was set at *p* < 0.05.

## Results

Athletes with the following characteristics participated in this study: age 34 ± 10 years, height 173 ± 10 cm, body mass 62 ± 7 kg, VO_2peak_ 2.23 ± 0.56 L·min^−1^, peak heart rate 181 ± 8 beats·min^−1^ and average weekly training volume 11 ± 5 h. Time-trial data with regards to performance, physiology and fatigue are presented in Table 1.

**Table 1:** **Physiological time-trial data**.

**Distance**		**400 m**	**800 m**	**5000 m**
Time [s]		61 ± 14	127 ± 36	840 ± 318
$\dot{\text{V}}$O_2_ Absolute [L·min^−1^]		2.15 ± 0.34	2.11 ± 0.34	2.03 ± 0.38
Relative to peak [%]		95.9	94.2	90.3
Heart rate Absolute [beats min^−1^]		169 ± 12	171 ± 10	178 ± 17
Relative to peak [%]		93.4	94.5	98.3
Blood lactate concentration at test end [mmol·L^−1^]		6.7 ± 3.0	8.1 ± 3.0	7.5 ± 3.9
Visual analog scale				
Physical fatigue [%]		77 ± 0.1	71 ± 0.2	69 ± 0.1
Respiratory fatigue [%]		70 ± 0.3	67 ± 0.3	57 ± 0.3

$\dot{V}$O_2_ : oxygen uptake

**Figure 1:**
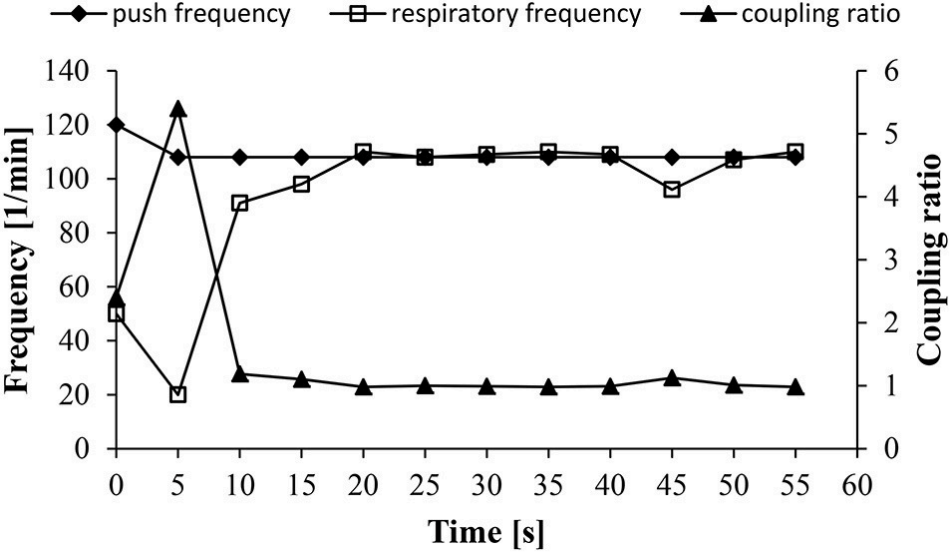
**Typical 1:1 coupling ratio between push frequency and respiratory frequency over 400 m after the initial starting phase**.

**Figure 2:**
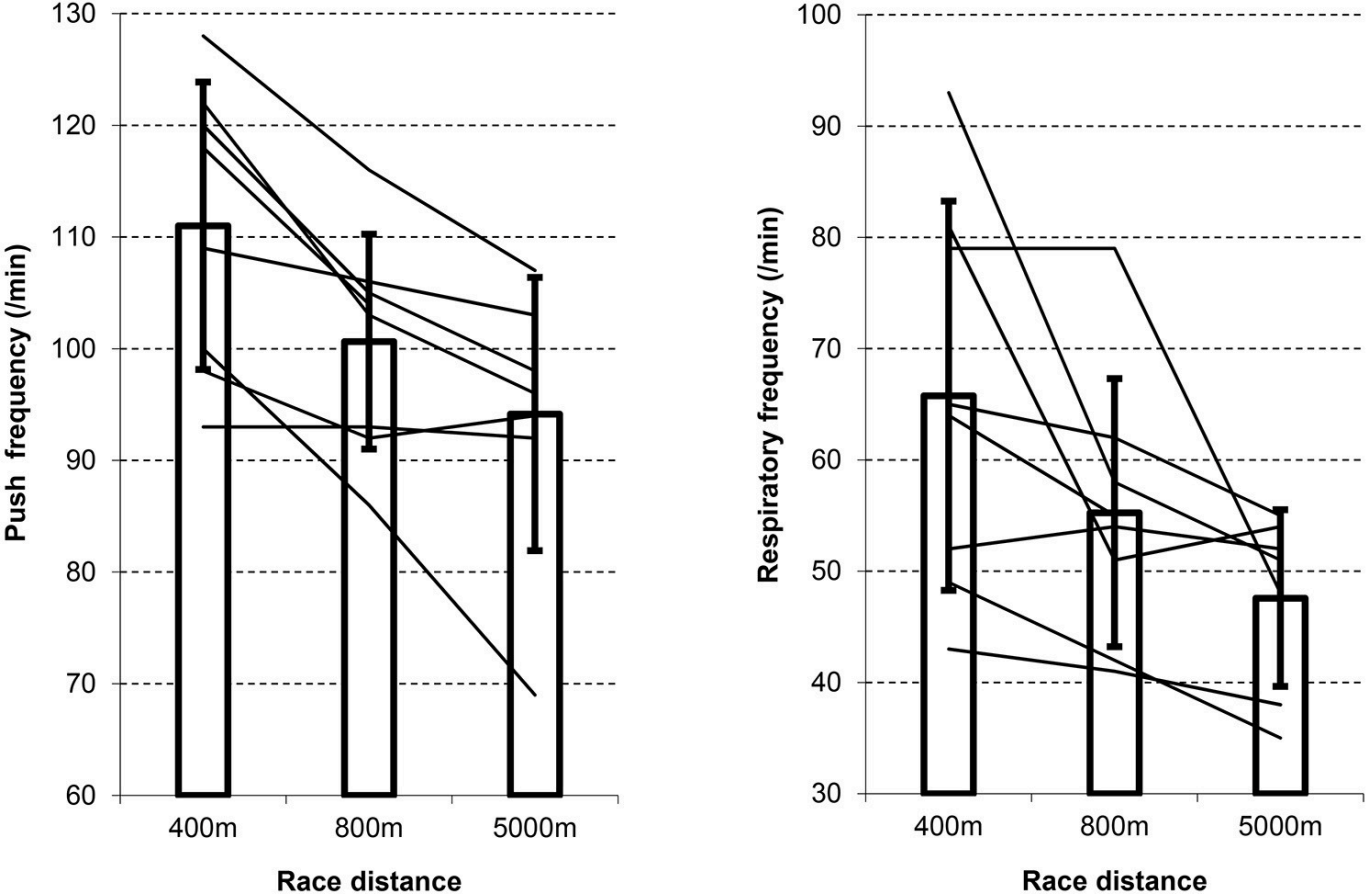
**Average and individual push and respiratory frequencies for the different race distances**. Note that average push and respiratory frequencies differed significantly (*p* < 0.05) between the different race distances.

Four different coupling types, namely 1:1, 2:1, 3:1, and a 1:1/2:1 alternating type, were identified. For each participant a predominant coupling ratio could be found for every distance covered (Table 2). A typical example of a 1:1 coupling ratio of a participant over the 400 m distance is shown in Figure 1. Over the 800 and 5000 m distance the most common coupling type was 2:1, whereas over the 400 m a 1:1/2:1 alternating coupling ratio was predominant. Please note that this alternating coupling type was in fact a feature and no artifact due to an unstable coupling one might expect during fast locomotion common for sprint exercise. Respiratory and push frequencies decreased with increasing race distance and were significantly different between the different race distances (Figure 2).

**Table 2:** **Predominant coupling types for the different race distances covered**.

**Participant**	**400 m**	**800 m**	**5000 m**
1	2:1	1:1/2:1	2:1
2	1:1/2:1	2:1	2:1
3	1:1	2:1	2:1
4	1:1/2:1	2:1	1:1/2:1
5	3:1	1:1/2:1	2:1
6	2:1	2:1	2:1
7	1:1/2:1	1:1	1:1/2:1
8	1:1/2:1	2:1	drop out

1:1/2:1 indicates a coupling type alternating between 1:1 and 1:2.

**Figure 3:**
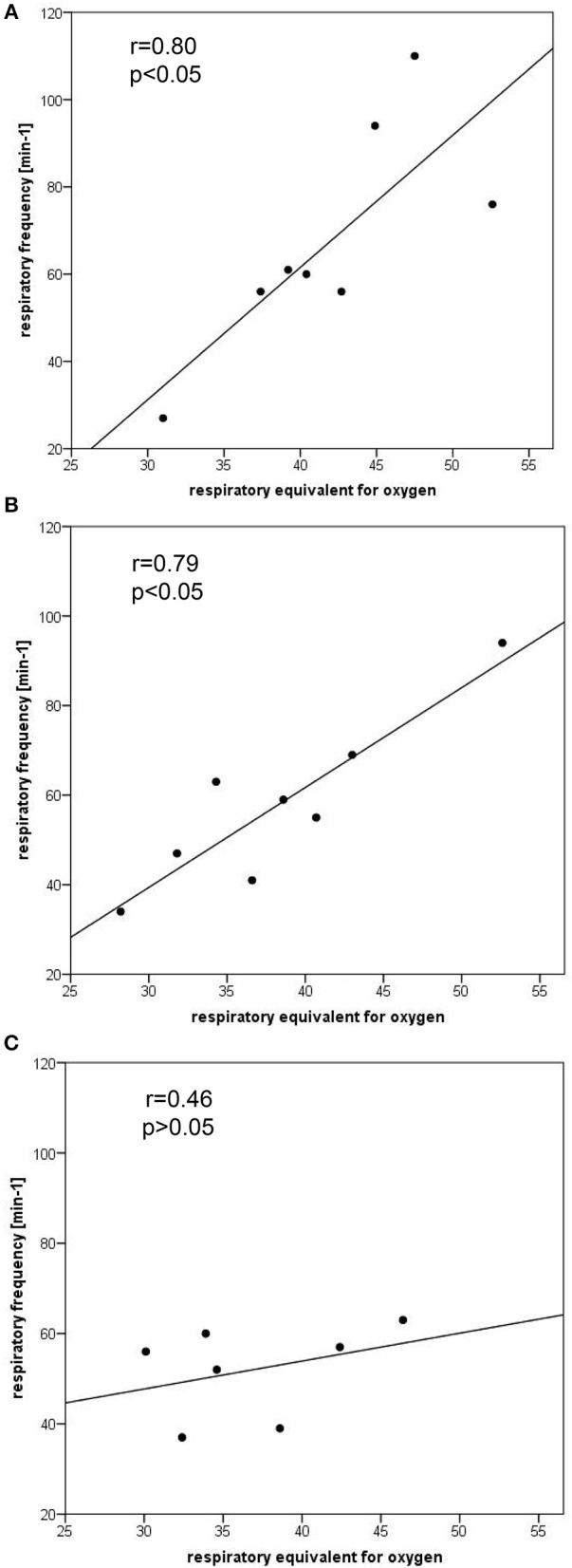
**Correlation plots comparing the ventilatory equivalent for oxygen vs. respiratory frequency for the 400 m (A), 800 m (B), and 5000 m (C) time trials**.

Significant correlations between respiratory frequency and the respiratory equivalent for oxygen were found for the 400 (*r* = 0.80, *p* < 0.05) and 800 m (*r* = 0.79, *p* < 0.05) but not for the 5000 m trial (*r* = 0.46, *p* > 0.05; Figure 3). Highly significant negative correlations were found between respiratory frequency and tidal volume for the 400 (*r* = −0.92, *p* < 0.01) and 5000 m *r* = −0.88, *p* < 0.01) but not for the 800 m (*r* = −0.63; *p* > 0.05) trial.

## Discussion

The main finding of the study was the existence of a LRC strategy with clearly identifiable coupling types in highly trained wheelchair racing athletes over the distances of 400, 800, and 5000 m. We found different coupling types (1:1, 2:1, 3:1, and alternating 1:1/2:1), changing individually depending on race distance. The overall predominant coupling type was a 2:1 ratio between push and respiratory frequency, which was predominantly found with increasing race distances. One likely explanation for this trend toward less frequent breathing per push with increasing race distance is the lower average speed over the longer distances, resulting in lower push frequencies and also a lower burden on the cardiovascular system (Goosey and Kirk, 2003). As a consequence, an economisation of the wheelchair push takes part (Goosey et al., 2000) leading to a reduced respiratory frequency. Our data further showed a lower respiratory frequency at higher tidal volumes, which suggests a more efficient respiration. The significant relationship between respiratory frequency and the respiratory equivalent for oxygen found in our study supports this assumption. Our data are further supported by previous research (Veeger et al., 1989; Vanlandewijck et al., 1994), demonstrating a relationship between speed and push frequency during steady-rate wheelchair propulsion exercise. Studies on daily wheelchair locomotion by MacDonald et al. (1992) refute the appearance of LRC during wheelchair propulsion, whereas Fabre et al. (2005) suggest the occurrence of LRC independent of arm movement frequency with no impact on improved economy of locomotion. This partly contradicts the present findings, however, one has to keep in mind that our study investigated elite wheelchair racing athletes reaching high race speeds and push rates of over 120 pushes·min^−1^. Additionally, the sitting position and hand rim size in a race chair markedly differ from the position in a daily wheelchair, which might have an impact on the LRC pattern. These differences make a direct comparison of our data with the above mentioned results from other studies difficult.

In accordance with data from experienced wheelchair users (Amazeen et al., 2001), we also found the 2:1 coupling ratio as the predominant coupling type in trained athletes. However, coupling ratios as high as 4:1 as previously reported (Amazeen et al., 2001), were not found in the present study. This may be due to differences in equipment (daily vs. race wheelchair), propulsion intensity (moderate vs. high intensity exercise) or training status (moderately trained vs. elite athletes). To expand on this issue of differences in wheelchair-user configurations, athletes can change the hand-rim size to suit the wheelchair racing distance, the handrim diameter being the equivalent to gears on a bicycle (Costa et al., 2009). Typically, sprinters would select smaller hand-rims since larger hand rims have a longer lever arm which could be a limiting factor at higher speeds (Costa et al., 2009). That said, the hand rim diameters used by our participants varied between 0.36 and 0.41 m, yet they were the same for individuals between trials. This may explain why the coupling ratios differed for seven of the eight athletes across the race distances—the limitation of a constant handrim size may have been compensated by alterations in the LRC. Further studies may investigate whether athletes settle for a preferred coupling ratio across race distances when given the choice of using a race distance dependent hand-rim. With regards to applicability of the present findings into an elite sport context, a particular strength of this study lies in the fact that the VO_2peak_ (2.23 ± 0.56L.min^−1^) measured was comparable if not greater to the work of similar athletic trained populations (Tolfrey et al., 2001; Goosey-Tolfrey and Tolfrey, 2004; Bernardi et al., 2010; Perret et al., 2012).

The present findings indicate that an efficient LRC strategy might play an important role in optimizing exercise performance in wheelchair athletics. However, final inferences about implications for efficiency of breathing on performance can't be made based on the present data and further studies are needed to elucidate this issue in more detail. In general, the importance of respiratory muscles is not limited to the respiratory system: respiratory muscles working at higher intensities can directly affect performance by reducing blood flow to working muscles (Harms et al., 1997). Delaying this metaboreflex by respiratory muscle training (Witt et al., 2007) or by reducing the load of respiratory muscles by an appropriate breathing or LRC strategy may therefore directly benefit performance. An efficient coupling strategy may even be more important during upper body exercise (e.g., during wheelchair propulsion) as respiratory muscles of the rib cage are concomitantly involved in ventilatory and non-ventilatory functions (Celli et al., 1988). Therefore, respiratory muscle training might be a future approach to either delay respiratory fatigue directly or indirectly and influence LRC efficiency in wheelchair athletes. Beside this, it is still unclear what determines differences in push strategies and how they are affected by different race durations and speeds or how this might influence individual LRC strategies. Thus, future studies should for example compare the impact on performance of self-chosen LRC vs. prescribed LRC strategies.

## Conclusions

We suggest that the selection of an appropriate LRC strategy might help to optimize wheelchair racing performance. However, final inferences about implications for efficiency of breathing on performance can't be made based on the present data. Additionally, one has to be aware that changing an individual LRC strategy might be challenging and require a considerable amount of coaching and training. Further studies are needed to determine the most efficient coupling types for different racing distances, how they may be influenced by adjustments in the wheel and handrim size and spinal cord injury lesion levels, and what training toward an optimal LRC strategy may entail.

## Author contributions

CP substantially contributed to the conception, design, and interpretation of the work, was involved in drafting the work, approved the final version to be published and agrees to be accountable for all aspects of the work. MW highly contributed to the design, acquisition and analysis of data, revised the work critically for important intellectual content, approved the final version to be published and agrees to be accountable for all aspects of the work. CL was substantially involved in the analysis and interpretation of data for the work, critically revised the manuscript for important intellectual content, approved the final version of the manuscript and agrees to be accountable for all aspects of the work. VG highly contributed to the interpretation of data, critically revised the manuscript for important intellectual content, made the final approval of the version to be published and agrees to be accountable for all aspects of the work in ensuring that questions related to the accuracy or integrity of any part of the work are appropriately investigated and resolved.

### Conflict of interest statement

The authors declare that the research was conducted in the absence of any commercial or financial relationships that could be construed as a potential conflict of interest.
